# Transmission of Airborne Bacteria across Built Environments and Its Measurement Standards: A Review

**DOI:** 10.3389/fmicb.2017.02336

**Published:** 2017-11-29

**Authors:** So Fujiyoshi, Daisuke Tanaka, Fumito Maruyama

**Affiliations:** ^1^Department of Microbiology, Graduate School of Medicine, Kyoto University, Kyoto, Japan; ^2^Graduate School of Science and Engineering, University of Toyama, Toyama, Japan; ^3^JST/JICA, Science and Technology Research Partnership for Sustainable Development Program (SATREPS), Tokyo, Japan

**Keywords:** airborne bacteria, holobiome, built environments, outdoor environments, bacterial communities, bacterial concentration, multiscale interaction

## Abstract

Human health is influenced by various factors including microorganisms present in built environments where people spend most of their lives (approximately 90%). It is therefore necessary to monitor and control indoor airborne microbes for occupational safety and public health. Most studies concerning airborne microorganisms have focused on fungi, with scant data available concerning bacteria. The present review considers papers published from 2010 to 2017 approximately and factors affecting properties of indoor airborne bacteria (communities and concentration) with respect to temporal perspective and to multiscale interaction viewpoint. From a temporal perspective, bacterial concentrations in built environments change depending on numbers of human occupancy, while properties of bacterial communities tend to remain stable. Similarly, the bacteria found in social and community spaces such as offices, classrooms and hospitals are mainly associated with human occupancy. Other major sources of indoor airborne bacteria are (i) outdoor environments, and (ii) the building materials themselves. Indoor bacterial communities and concentrations are varied with varying interferences by outdoor environment. Airborne bacteria from the outdoor environment enter an indoor space through open doors and windows, while indoor bacteria are simultaneously released to the outer environment. Outdoor bacterial communities and their concentrations are also affected by geographical factors such as types of land use and their spatial distribution. The bacteria found in built environments therefore originate from any of the natural and man-made surroundings around humans. Therefore, to better understand the factors influencing bacterial concentrations and communities in built environments, we should study all the environments that humans contact as a single ecosystem. In this review, we propose the establishment of a standard procedure for assessing properties of indoor airborne bacteria using four factors: temperature, relative humidity (RH), air exchange rate, and occupant density, as a minimum requirement. We also summarize the relevant legislation by country. Choice of factors to measure remain controversial are discussed.

## Introduction

Human health is strongly associated with the balance of microbial communities (Blaser, 2014). The human microbiome provides protection from skin pathogens (Grice and Segre, 2011), aids digestion, supplies nutrients, and activates the immune system (Eckburg et al., 2005; Walia et al., 2014; Adar et al., 2016). A balanced microbial community is more resilient and is better able to protect against pathogen invasion. Dysbiosis impacts the overall stability of a microbial community, leaving the host susceptible to infection and inflammation, with some recent reports showing a link between dysbiosis and immune disorders (Honda and Littman, 2012).

For various macroscale ecosystems, such as coral reefs, it is well established that greater biodiversity increases the efficiency by which ecological communities can use essential resources. Coined in 1992, the term *holobiont* originally defined host-microbe symbioses (Mindell, 1992). Corals establish a symbiotic relationship with specific zooxanthellae in their surrounding environments, from which they obtain various nutrients. Corals can also use metabolites from microorganisms and cyanobacteria (Thompson et al., 2014; Cardini et al., 2016). In addition to supplying nutrients, these symbiotic microorganisms are involved in the implantation and development of their host, and provide resistance to pathogens (Thompson et al., 2014).

It is essential to understand holobiotic systems for managing human health and disease, because the human microbiome is associated with health outcomes (Postler and Ghosh, 2017). The bacterial composition is readily altered as a result of dietary changes, use of antibiotics, infection, and environmental factors (Eckburg et al., 2005; Walia et al., 2014; Adar et al., 2016). In particular, built environments, where people spend up to 90% of their time, are likely to influence human health (Klepeis et al., 2001). Each day, the air inhaled by a human typically contains 10^6^ airborne microorganisms (Mandal and Brandl, 2011). Some of these microorganisms cause pneumonia (e.g., non-tuberculous mycobacteria (NTM), *Legionella* and *Mycoplasma* species), asthma, or allergies (Dannemiller et al., 2016; Montagna et al., 2016; Nishiuchi et al., 2017). More generally, it has been argued that childhood exposure to reduced levels of microbial diversity in and around homes may partially explain the rise in the incidence of allergies and autoimmune disorders in many developed countries (Fujimura et al., 2014; Fang et al., 2016; Man et al., 2017). In high population density indoor environments, such as correctional facilities (Hoge et al., 1994), military training centers (Brundage et al., 1988), and dormitories, human-to-human transmission often occurs. Furthermore, airborne transmission of bacteria in health care facilities can cause nosocomial infections (Schaal, 1991). The link between mental health and the microbiome of the built environment is discussed by Hoisington et al. (2015). Therefore, identifying the bacteria present in indoor environments is critically important for human health.

**Figure 1:**
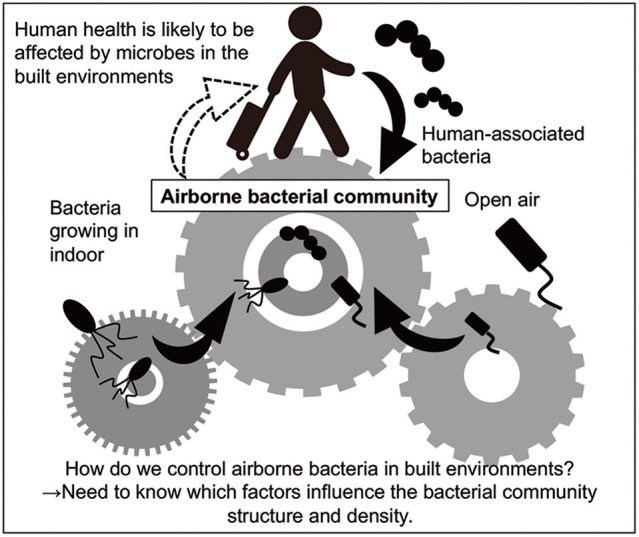
Overview of the present review. Human health is probably affected by bacteria in built environments, because people spend approximately 90% of their lives there. Humans and outdoor air are likely to be the major sources of airborne bacteria as well as bacteria growing in indoors (Burrows et al., 2009; Fahlgren et al., 2010; Bowers et al., 2013).

**Figure 2:**
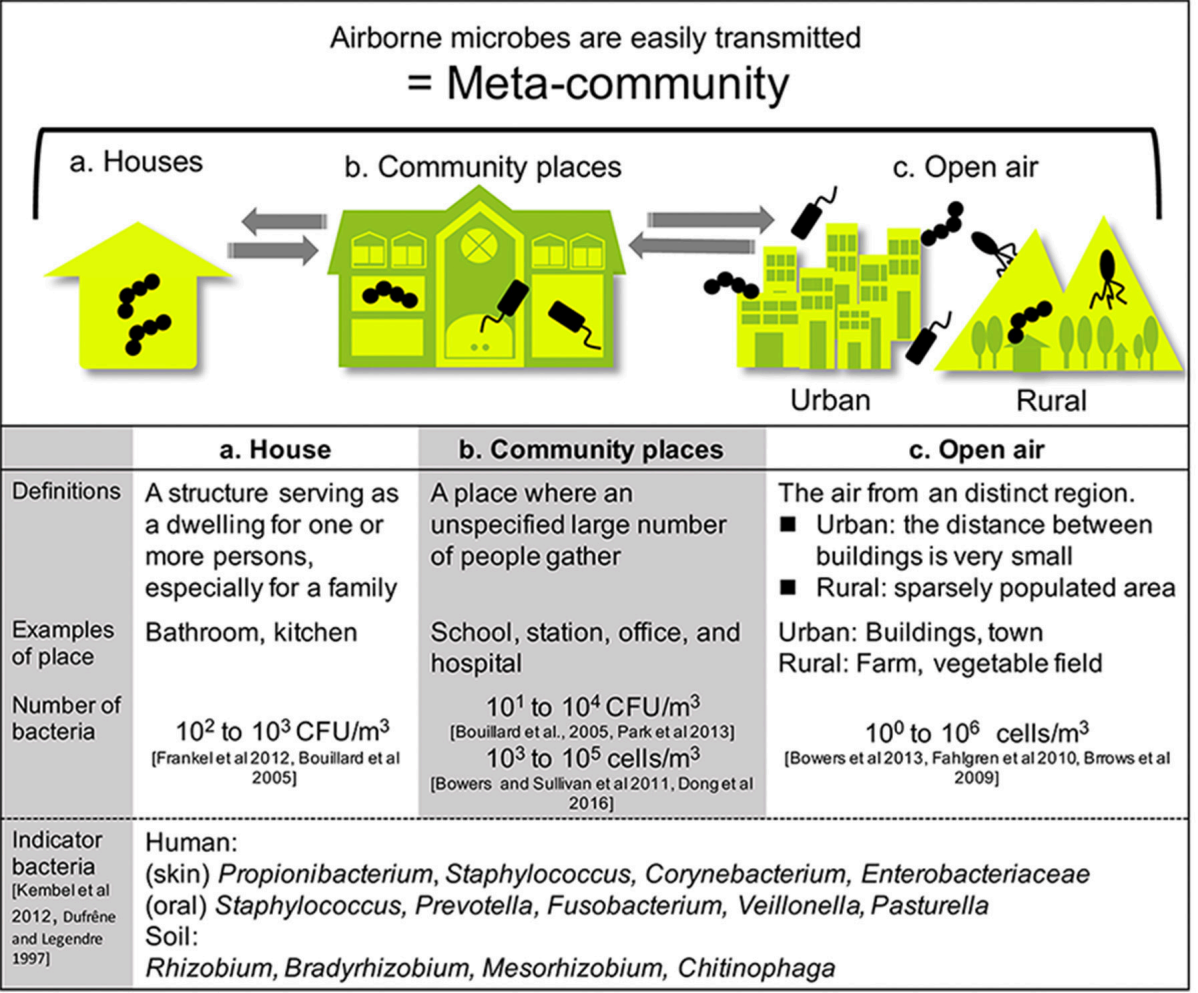
Built environments were classified into two areas: houses and community spaces. People living and working in such spaces constantly interact with microbes. Airborne microbes also occupy a wide range of built environments. Each type of space is connected by air, including open-air environments.

This review describes the airborne bacteria and its likely sources in built environments (Figures 1, 2). (Many comprehensive reviews of airborne fungi already exist; Prussin and Marr, 2015; Adams et al., 2016 as a result, our review focusses on bacteria).

We review the likely factors affecting bacterial concentration and community fluctuations, focusing on temporal variations (temporal and seasonal) and multiscale interactions. The Genomic Standards Consortium (GSC, http://gensc.org) has introduced a system for describing the environment from which a biological sample originates, described as “environmental packages.” They provided a package for bacterial sequences within built environments (MIxS-BE (Minimum Information about any (x) Sequence-Built Environment): http://gensc.org/index.php?title=MIxS_extensions) in 2014 (Glass et al., 2014). In this MIxS-BE package, 26 metadata package terms (e.g., carbon dioxide, ventilation type, filter type, light type etc.) are provided as well as MIxS-air environmental package terms. These metadata collections could improve indoor microbial community characterization (Ramos and Stephens, 2014); however, it is difficult to collect data on the full set of factors in a typical sampling situation. We therefore, propose four factors (temperature, RH, air exchange rate, and occupant density) that as a minimum should be routinely measured to monitor and control airborne bacteria in built-up environments. We also summarize the relevant legislation by country.

## Sources of bacteria

Airborne bacteria can be treated more effectively if their origin is known. In this section, we summarize the sources of bacteria in two main areas of built (house and community places), and open-air environments (as outlined in Figure 2). Many fungi grow in built environments such as water-damaged homes, schools, and daycare centers, creating severe sanitary problems and potentially being responsible for health issues in humans (Mendell et al., 2011). The impact of airborne fungi on human health means that many state-of-the-art reviews have been published on this topic (Rao et al., 1996; Dillon et al., 1999; Portnoy and Jara, 2015). In contrast, far fewer reviews have been published on airborne bacteria in built environments. Therefore, we have focused on airborne bacteria in built environments in this review.

### Bacteria in houses

**Figure 3:**
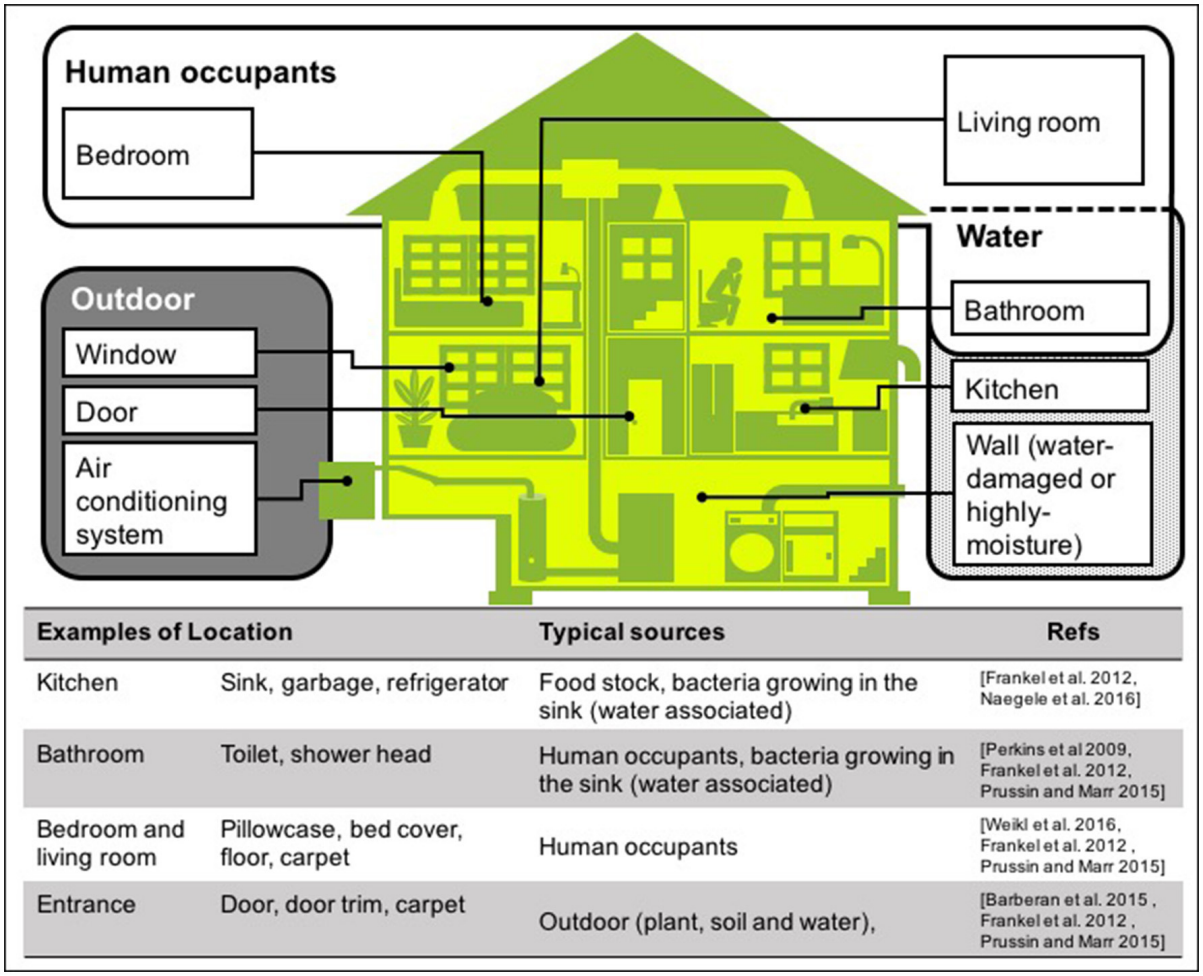
Typical sources of airborne bacteria in a house. Three sources are shown: human occupants (white area), water (dotted area), and outdoors (gray area). Note that these are the groups we have used in this room, but other studies have presented alternatives (Dunn et al., 2013; Prussin and Marr, 2015; Sun et al., 2016).

There are many sources of bacteria found in houses, although the majority of house microbe studies have collected samples using surface swabbing as a proxy for integrated airborne bacterial sampling. Here we describe surface bacteria as inferred airborne bacteria, but for detail of the precise sampling method for each study, see Table 1 “enumeration technique.” The main locations and sources of bacteria in houses are shown in Figure 3. In this review, we discuss the three most common sources: human occupants, water, and the outdoor environment, because in our current review, we aim to determine what environmental factors should be measured to understand indoor airborne bacterial communities and concentrations.

**Table 1:** Changes in microbial community composition, and the typical sources that influence these changes, from a temporal and seasonal perspective.

**Area (detail)**	**Typical sources**	**Measurement period**	**Measured parameters and other relevant information**	**Enumeration technique air or surface sampling (bacterial conc. CFU/m^3^)**	**Dominant bacteria**	**References**
**INDOOR AIR**						
Biosphere (plant cabin, waste treatment room, bathroom, kitchen and living room, and bed room)	Humans, plants, pets	105 days	—	Air sampling Culture (fungi and bacteria colony densities of the plant cabin were controlled less than 2,500 CFU/m^3^) Non-cultivation (MiSeq)	Phylum *Proteobacteria, Firmicutes*, Cyanobacteria	Sun et al., 2016
Store (HVAC filter)	Humans	Two seasons (Summer and Winter) for 1 month each	Season Air exchange rate (l/h) Volume (m^3^) Occupant density (/100 m^2^)	HVAC filter dust Non-cultivation (454 GS FLX)	Family Bacillaceae, Corynebacteriaceae	Hoisington et al., 2016
Hospital (lobby and included the waiting areas and corridors, and air ducts)	N/A (relatively stable)	6 months	Temperature and RH were maintained at 21°C and 40%, respectively.	Air sampling Non-cultivation (HiSeq)	Order Bacillales, Lactobacillales, Propionibacteriales, Micrococcales, Actinomycetales, Corynebacteriales, Pseudomonadales, Xanthomonadales	King et al., 2016
Hospital (patient rooms and nurse stations)	Humans	1 year	Temperature (°C) RH (%) Humidity ratio (g_w_/kg_da_) Illuminance (lx) IR beam-break (counts/5 min) CO_2_ (ppm) Differential pressure (Pa)	Surface sampling, and air sampling via ultraviolet-sterilized MERV 7 filter medium placed over the return air grilles in the patient rooms Non-cultivation (HiSeq and MiSeq)	Family Corynebacteriaceae, Streptococcaceae, Staphylococcaceae, ^*^human skin-associated bacteria	Lax et al., 2017
Hospital (lobby)	Season Human	Two seasons (Summer and Winter)	Temperature (°C) RH (%) CO_2_ (ppm) Number of bed Building age Area of lobby Service hours (8:00–18:00) After service hours (18:00-24:00) Cleaning time ^*^After lobby service hours, the HVAC system was not operating.	Air sampling Culture (7.2 × 10^2^)	—	Park et al., 2013
Office (two buildings located in small towns in central Finland)	Building trends	Two seasons (Summer and Winter)	Building information (age, structure, usage, and willingness to participate) Weather information Average temperature (°C) Number of persons	Dust sampling Non-cultivation (ABI3700 automated DNA sequencer)	Family Corynebacteriaceae, Propionibacteriaceae, Streptococcaceae, Staphylococcaceae, Peptostreptococcaceae, Lactobacillaceae	Rintala et al., 2008
University libraries	Humans	1 month and twice a day	Area (m^2^) Cubature (m^3^) Number of persons	Air sampling Culture (367–2,595)	Family Micrococcaceae, Streptococcaceae, Staphylococcaceae	Stryjakowska-Sekulska et al., 2007
Classroom (The schools were in five cities in four countries, from three continents.)	Humans (14 × 10^6^ bacterial cells/(person × h))	Occupied sampling: approximately 25 total hours Unoccupied sampling: about 60 h	Temperature (in/out) RH (in/out) CO_2_ (ppm) Volume (m^3^) Floor area (m^2^) Occupant density (person/m^2^) Number of windows Operable windows (Yes/No) Mechanical ventilation (Yes/No) ^*^Five of the six classrooms relied on natural ventilation via windows only, and one classroom was mechanically ventilated that supplied 100% outdoor air into the class room.	Air sampling Non-cultivation (no phylogenetic analysis) Number concentrations of total fungi and bacteria were determined by 18S and 16S qPCR.	—	Hospodsky et al., 2015
Classroom	Humans (37 × 10^6^ bacterial genome copy number/(person × h))	Four days in September or November	Vacant/occupied Cubature (m^3^) Ventilation system (HVAC) Air exchange rate (l/h) CO_2_ (ppm) Temperature (°C) RH (%) Number of persons Size distribution of microorganisms ^*^All windows and doors were closed and conditioned air was delivered by the building HVAC system.	Air sampling Non-cultivation (454 GS FLX) Human occupancy was associated with substantially increased airborne concentrations of total particles, bacterial genomes, and equivalent fungal genome. Indoor airborne particulate matter during occupancy was enriched in bacteria	Order *Indoor occupied* Actinomycetales, Xanthomonadales, Sphingomonadales *Outdoor occupied* Rhizobiales, Flavobacteriales, Actinomycetales	Qian et al., 2012
**OPEN-AIR ENVIRONMENT**						
City (one small town and three metropolitan areas in USA)	Season	6 weeks in two seasons (summer and winter)	Temperature (°C) Weather (sunny, snow etc.) Wind speed (kph) Wind direction	Air sampling Non-cultivation (454 GS FLX) Total bacterial^*^ concentrations 10^3^ – 10^5^ cells/m^3^ ^*^Measured via flow cytometry The bacterial concentrations observed in air is reduced during winter	Order ^*^Strong seasonal patterns in bacterial community *Summer* Pseudomonadales, Burkholderiales, Rhizobiales, Sphingomonadales ^*^In summer, it is likely to be derived from both soil and leaf source *Winter* Bacteroidales, Clostridiales, Fusobacteria ^*^In winter, dog feces are likely the dominant sources of bacteria	Bowers et al., 2011b
City (Building, paved lots, and grass field in University of Colorado campus, Boulder, USA)	Humidity (not particularly strong)	8 days	Temperature (°C) RH (%) Wind speed (km/h) Wind direction Pressure (hPa) Solar radiation (W/m^2^)	Air sampling Non-cultivation (clone library analysis, ABI 3730 DNA Analyzers)	Family Flavobacteriaceae, Flexibacteraceae, Pseudoonadaceae, etc. ^*^Phylogenetically diverse	Fierer et al., 2008
City (coastal region of Qingdao, Chinna)	Weather •On foggy days, temperature and O_3_ are negatively correlated with microbial concentrations •On hazy days, PM_2.5_, SO_2_, NO_2_, CO, and air quality index are positively correlated with microbial concentrations	1 year	Temperature (°C) RH (%) Wind speed (m/s) SO_2_ NO_2_ CO O_3_	Air sampling Total microbial concentrations (direct count) *Non-hazy days* 10^3^-10^5^ cells/m^3^ *Hazy days* 7.09 × 10^5^ cells/m^3^ *Foggy days* 9.00 × 10^5^ cells/m^3^	–	Dong et al., 2016
Subway (inside the trains, boarding platform and ticket office)	No important factors	4 months in winter	Temperature (°C) RH (%) Air-conditioning was functioning or not	Air sampling Non-cultivation (454 GS FLX)	Family Methylobacteriaceae, Chitinophagaceae, Bradyrhizobiaceae	Triado-Margarit et al., 2017
Top of Mt. Werner in northern Colorado, USA	Season (both bacterial community and concentration)	Spring, Summer, Autumn, Winter	Temperature (°C) RH (%) Wind speed (km/h) Wind direction	Air sampling Non-cultivation (454 GS FLX) Total bacterial^*^ concentrations 10^3^ – 10^5^ cells/m^3^ ^*^Measured via flow cytometry	Order or family *Spring* Sphingomonadaceae, Actinomycetales *Summer* Pseudomonadaceae, Actinomycetales, *Autumn* Bacillales, Actinomycetales, *Winter* Actinomycetales, Moraxellaceae,	Bowers et al., 2012

* Authors' comments.

One of the sources is human occupants. Most of the bacteria found in houses originate from the skin and intestinal tracts of humans, and include species belonging to the bacterial phyla *Proteobacteria, Actinobacteria, Firmicutes*, and *Micrococcus* (Prussin and Marr, 2015; Peccia and Kwan, 2016). When humans move to a new dwelling, the microbial communities on the surfaces of the new home quickly correlate with those on the skin of the new occupants (Lax et al., 2014). Bacteria from the oral cavity and feces are predominantly found on pillowcases and toilet seats, respectively (Dunn et al., 2013).

The second major source is water, which disseminates bacteria through the home via showers, taps, and toilets (Perkins et al., 2009; Ichijo et al., 2014). Bacterial growth in buildings may also be facilitated by water leaks, floor flooding, and in relevant areas, the rainy season. Once moisture has accumulated on building surfaces, biocontaminants may proliferate on surfaces and then be dispersed as bioaerosols (Fabian et al., 2005). Flooding caused by natural disasters may also promotes mold growth and dispersed of bioaerosols. For example, the tsunami caused by the Great East Japan Earthquake in 2011 damaged seaside cities. In flood-damaged homes with flooding above floor level, 63.3% of respondents (*N* = 256) reported problems after flooding. Mold growth was significantly associated with flooding height (Hasegawa et al., 2015). In the heavily water-damaged houses caused by Hurricanes Katrina and Rita in 2005, culturable fungi were significantly higher in moderately/heavily water-damaged houses (geometric mean 76,000 CFU/m^3^) than in mildly water-damaged houses (geometric mean 3,700 CFU/m^3^) (Rao et al., 2007).

The third source is the outdoor environment. Airborne bacteria enter houses via natural ventilation components such as windows and doors, while other environmental bacteria are transferred from shoes onto floors and carpets (Bouillard et al., 2005). Microbial communities found on floors and carpets probably reflect cultural differences. For example, in Japan, where shoes are removed before entering a room, the floor microbial communities are likely to be dominated by human skin bacteria rather than soil bacteria. Pets and plants are also important sources of indoor microorganisms. Closed artificial ecosystem experiments in China showed that food stocks in kitchens also disseminate airborne bacteria (Sun et al., 2016).

It should be noted that other researchers have used alternative lists and definitions of these sources for indoor airborne microbes. Sun et al. (2016) monitored airborne microbes in closed artificial ecosystems, and described the possible three sources within that system: (1) human bodies, animals, and plants; (2) microbial contamination during the process of assembling the experimental cabins; and (3) contamination from outside. According to Dunn et al. (2013), they selected nine locations in houses as standard sampling locations, based on their existence in virtually all homes: kitchen cutting board, kitchen counter, refrigerator shelf, toilet seat, pillowcase, exterior handle of the main door into the house, television screen, trim of an exterior door, and the upper door trim on an interior door. Prussin and Marr defined eight indoor bioaerosol sources in the built environment: humans; pets; plants; plumbing systems; heating, ventilation, and air-conditioning systems; mold; resuspension of settled dust; and outdoor air (Prussin and Marr, 2015).

### Community places

Similar to the house microbiome, bacterial communities in offices, classrooms, hospitals and other community spaces are dominated by human and soil bacteria (Hewitt et al., 2012; Hoisington et al., 2016). The main difference between houses and community places is the number of people using the area. People frequenting an area can change the microbial community composition of a space, and the number of people and types of human activities are positively correlated with the concentration of bacterial bioaerosols (Heo et al., 2017). To a lesser but significant extent, these factors also influence the composition of indoor bioaerosols (Adams et al., 2015).

Air-mixing is related to many factors: frequency of cleaning, the number of ventilation points, the location of the ventilation system, window positions, floor area, room and building heights, number of occupants, diffuser types, and air speeds and flows. As all of these factors are related to the architectural design of the indoor space, the relationships among ventilation systems, air ducts, exhaust fans, indoor air intake ports, and the microbiome are attracting increasing interest (Kembel et al., 2012; Yang et al., 2016; Zhou et al., 2016). Yang et al. (2016) calculated the fluence rate of the multiple ultraviolet germicidal (UR-UVGI) fixtures system and provide the simulation model, which was validated using the data reported by Rudnick et al. (2015). The results showed that the position of the UR-UVGI fixture near the outlet achieved the most efficient disinfection rate. Important mechanisms that remove bioaerosols from air include air exchange, deposition onto indoor surfaces, and active filtration. Nazaroff reviewed the dynamic processes that govern indoor concentrations and fats of biological particulate material (Nazaroff, 2016). He emphasized that bioaerosol behavior is strongly coupled to particle size (0.1–10 μm). Airborne microorganisms and sampling methods in various selected indoor locations were reviewed by Mandal and Brandl in 2011 (Mandal and Brandl, 2011).

### Open-air environments

The sources of airborne bacteria are more diverse in open-air environments than in indoor spaces, and include soil, water, plants, and insects. The composition of airborne bacterial communities is influenced by geographical variations such as landscape and land use. For example, crop harvesting significantly increases the number of airborne bacteria (Elin et al., 1984). In open-air environments, bacterial sources are difficult to identify because microorganisms are released and transported by various organisms and events. For example, one station that was flooded by Hurricane Sandy in the United States still resembled a marine environment 2 years later (Afshinnekoo et al., 2015). Although that study used a swab sampling method rather than an air sampler, such events may also affect airborne microbial communities.

### Relationships between surfaces and aerosol loading

Although bacterial sources differ in indoor and outdoor environments, human-associated and outdoor environmental bacteria dominate the bacterial communities in indoor environments. However, the majority of house microbe studies collected samples by surface swabbing as a proxy for integrated airborne bacterial sampling. The correlation between surfaces and aerosols, and the surface-aerosol interactions in indoor environments, require further investigation. In particular, the volume of particles deposited on and then detached from a particular surface is poorly understood. Proving the abovementioned interaction is important for assessing cleaning frequency and tracking the potential impacts of microbes on human health in indoor environments.

## Time-course analysis of bacterial community composition

In section Sources of Bacteria, we stated that the majority of bacteria in indoor environments can be derived from humans, but they depend on many other factors. In some cases, such as low occupant density and high air-change per hour, human sources are less influential than outdoor bacterial sources (Hospodsky et al., 2015). Therefore, to understand airborne bacteria in the built environment, it is necessary to analyze both bacterial communities and concentration, as well as environmental factors including human occupation.

### Number of humans affect the bacterial concentration but not the communities in built environments

Humans are surrounded by a vast bacterial ecology (Tyakht et al., 2013; Meadow et al., 2015; Metcalf et al., 2017). These bacteria originate from human skin, the oral cavity, and intestine, as well as from clothes. In university classrooms, domiciles, offices, health care facilities and other buildings, the concentration of microorganisms increased during the occupied periods and declined during vacant periods (Stryjakowska-Sekulska et al., 2007; Qian et al., 2012; Park et al., 2013; Hospodsky et al., 2015). According to Qian et al. (2012), all windows and doors were closed during the experiments, and a strong signal of human associated microbes was detected. In the subway systems of Barcelona and Hong Kong, the diversity of airborne microbes was not affected by commuters (Leung et al., 2014; Triado-Margarit et al., 2017). Taxonomic comparison of the microbes from the Hong Kong and New York City subway networks revealed that *Arthrobacter, Psychrobacter*, and *Enhydrobacter* were the predominant bacterial genera in both locations (Robertson et al., 2013). The bacteria possibly originate from the microbial communities of nearby outdoor and human sources. On the Mass Transit Railway (MTR) in Hong Kong, the bacterial diversity of the collected samples was time-dependent, being more diverse in the afternoons and evening than in the morning. In contrast, the diversity was apparently uninfluenced by commuter traffic (Leung et al., 2014). Airborne microbes around the MTR lines potentially reflect those of nearby outdoor locations, which include soil, water, and leaf-associated organisms (Leung et al., 2014). The point is that in closed-indoor environments, humans are the single main source of bacteria, so there are different bacterial compositions during occupied and vacant periods. In contrast, in open-built environments such as MTR, humans are one of many sources of bacteria, so human-associated bacteria are still present. Varying numbers of humans do not affect bacterial species composition but do affect bacterial concentration. Therefore, humans are likely to affect bacterial concentration but not influence the overall community diversity.

### Temporal and seasonal variations

**Figure 4:**
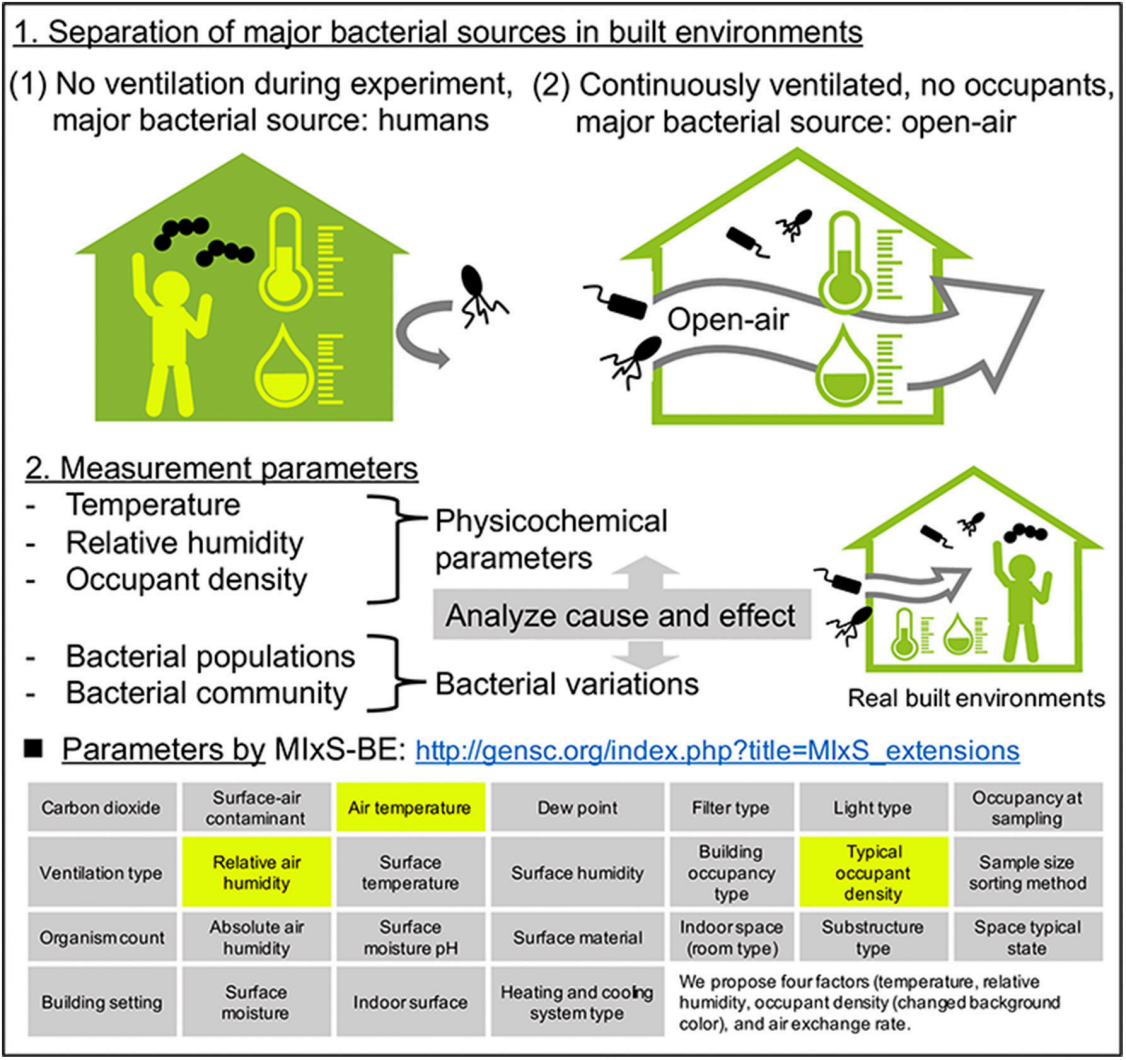
A plan for analyzing the causal relationship between seasonal bacterial variations and physicochemical parameters in built environments. The four factors (temperature, relative humidity, occupant density, and air exchange rate) are the minimum requirement for monitoring that we recommend.

Although bacterial communities reportedly vary with the seasons, the factors governing these seasonal variations remain unknown. This is due in part to the multiple bacterial sources in the experimental system. As described in section Number of Humans Affect the Bacterial Concentration but Not the Communities in Built Environments, indoor airborne bacterial communities are assumed to be mainly composed of human and outdoor environmental bacteria. During indoor air sampling, the microbial fluctuations are mainly correlated with human occupancy (Table 1). Moreover, they also depend on occupancy density and levels of human activity, such as running, walking, sitting, and talking (Qian et al., 2014; Adams et al., 2015; Meadow et al., 2015). However, over longer periods, occupants probably use natural ventilation (windows and doors), meaning that at least two different bacterial sources are mixed during the experiment (occupants and outdoor). Researchers Hospodsky et al. (2012), Dunn et al. (2013), and Meadow et al. (2014) together combine the fields of microbial ecology, building materials and architectural design, to understand microbial diversity and abundance within a building. To clarify the individual factors correlated with bacterial community changes, and indoor–outdoor bacterial interaction, the following experiment, originally conducted by Kembel et al. (2012), provides a good illustration. Researchers collected samples from outdoor air, indoor air from a mechanically ventilated room, and indoor air from a “naturally” ventilated room, simultaneously. The bacterial species related to humans and environments, such as water and soil, were used as an indicator of indoor bacterial community fluctuation (Dufrêne and Legendre, 1997). They showed that building attributes, specifically the source of ventilation air, airflow rates, relative humidity and temperature, were correlated with the diversity and composition of indoor bacterial communities. According to their study design, two control experiments are conducted (Figure 4):

1. Occupants remain, no ventilation during the experiment (e.g., no open windows)
2. No occupants, windows and doors are open during the experiment

The physicochemical factors outlined in section Measured Factors at the Time of Airborne Bacterial Sampling in Built Environments are then be measured in both systems. In system (1), the major bacterial source is the occupants. In this case, human-related bacteria would make up the entire bacterial community in the built environment, and we could analyze cause and effect between physicochemical factors and bacterial community composition. A good example of system (1) was studied by King et al. (2016) in hospitals, where they monitored airborne bacteria in hospital lobbies, waiting areas, corridors, and air ducts, with temperature and humidity maintained at 21°C and 40%, respectively. They found that the bacterial composition was relatively stable in hospital environments, suggesting that if there is one bacterial source (such as humans), and environmental factors are stable, the bacterial community would also be stable. It is important to note that this discussion concerns bacterial species composition, and not bacterial concentration.

In contrast, in system (2), open-air environmental bacteria would dominate the bacterial community. The problem in this case is that the effects of environmental conditions on airborne bacterial communities in open-air environments are not well understood, mainly because the bacterial sources are more diverse than in indoor environments. Airborne microbes in open-air environments are also influenced by factors such as wind strength and direction, humidity, temperature, and bacterial spore-forming cycles (Fierer et al., 2008). With separating major bacterial sources, analyzing causal relationships between physicochemical parameters and bacterial fluctuations could help us to clarify the factors governing seasonal variation of bacteria. It is interesting to note the factors that were measured when this study was originally conducted by Kembel et al. (2012): room size and the ventilation rate were measured and compared, allowing comparison of bacterial communities. Mixed effects models, source-sink analysis, and trajectory analysis also help us to understand indoor-outdoor bacterial interaction (Frankel et al., 2012; Miletto and Lindow, 2015).

## Meta-communities: relationships among the bacteria inhabiting indoor and outdoor areas

As shown in section Bacteria in Houses, bacterial communities of houses are dominated by human-associated and environmental bacteria. In these spaces, the number and composition of bacterial residents are usually stable, and are mainly altered by ventilation (Table 2). In contrast, microbial communities in community spaces are difficult to trace, as they are dynamically altered by the large amount of foot traffic and the high degree of ventilation. The interaction between indoor and outdoor air has previously been evaluated. The type of ventilation system (artificial or natural), number of ventilation points, and the placement of the ventilation system all affected the concentrations of airborne particles in the indoor and outdoor air samples (Sattar et al., 2016). As seen in section Community Places, many bacteria in community spaces are sourced from occupant behavior, such as opening windows, which also affects the indoor air quality.

**Table 2:** Interactions between indoor and outdoor environments, and the influence of outdoor land-use type on other areas.

**Area (detail)**	**Typical sources and causes**	**Measurement period**	**Measured parameters and other information**	**Enumeration technique air or surface sampling (bacterial conc. CFU/m^3^)**	**Dominant bacteria**	**References**
**INDOOR AND OUTDOOR**						
House and outdoor (Indoor: upper door trim on an interior door in the main living area of the home, outdoor: upper door trim on the outside surface of an exterior door)	Humans	N/A	Questions concerning building characteristics (address, location etc.) Residents (number, age, number of bedrooms) Use of insecticides, female/male ratio, number of pets and house plants	Surface sampling by volunteers Non-cultivation	Family *Indoor* Corynebacteriaceae, Streptococcaceae, Lactobacillaceae *Outdoor* Corynebacteriaceae, Streptococcaceae, Lactobacillaceae	Barberán et al., 2015 See also their website: http://robdunnlab.com/projects/
House and outdoor air (dust form living room floors)	Outdoor (plants) For bacteria, the rapid and strong change in community variation and reduced diversity during the spring.	301 days	Occupant behavior (ventilation, having pets, and smoking, etc.) Building characteristics Outdoor pollution Vegetation Urbanization	Dust sampling with vacuum cleaners Non-cultivation (ABI 3730 capillary sequencer)	— ^*^Fingerprinting technique used did not allow the identification of microbial taxa	Weikl et al., 2016
House and outdoor (kitchens, bedrooms, living rooms, and bathrooms of the homes, and outdoors in Danish)	Seasons Temperature and air exchange rates were negatively associated with bacteria. For bacteria, indoor concentrations were always higher indoors than outdoor.	1 year	Season (temperature, RH) Building age Number of persons (adults/children) Number of pets Floor area Number of rooms Ventilation type (natural/mechanical) Known moisture/fungal problems ^*^All windows of the homes were kept closed during sampling, and external and internal doors of the homes were kept closed during sampling	Dust sampling with vacuum Culture Spring (2,165)-Summer (240)	—	Frankel et al., 2012
House and compost (apartments and individual houses)	N/A (Indoor composting has no significant effects on the bioaerosol levels and the surface microbes beyond 0.5 m from the waste bin)	12 months	Thirty-four questions relating to: •the dwelling itself (address, location, etc.) •the kitchen (ventilation, floor, and wall coverings) •the residents (number, age, number of smokers) composting practices of the residents (waste bin, waste type)	Air sampling, electrostatic dust sampling, and swipe Culture ^*^Actinomyces, with waste bins: 8 CFU/m^3^, without 8 CFU/m^3^	^*^Used fungi and actinomyces selection medium *Laceyella sacchari, Saccharomonospora viride, Thermoactinomyces vulgaris*	Naegele et al., 2016
Classroom and outdoors	Ventilation, occupancy, and outdoor air sources	August 1–11	Cubature (m^3^) Number of persons Ventilation system and time ^*^Indoor rooms (night-flushed/ non night-flushed) received a combination of unfiltered outdoor air and mechanically ventilated, filtered air.	Air sampling Non-cultivation (MiSeq)	Family *Indoor* Corynebacteriaceae, Staphylococcaceae, Moraxellaceae *Outdoor* ^*^Substantial overlap between indoor and outdoor communities; 88 of the 100 most common indoor OTUs were also among the 100 most common outdoors.	Meadow et al., 2014
Community places and outdoors (daycare centers and elementary schools, the rear of the classroom)	Humans	August 19 to October 24	Building characteristics (size, location) Number of children Ventilation (window) Temperature (°C) RH (%) ^*^Ventilation was provided through windows.	Air sampling Non-cultivation (Roche/454 GS Junior)	Family *Indoor* Micrococccaceae, Staphylococcaceae, Streptococcaceae *Outdoor* Methylobacteriaceae, Streptomycetaceae, Micrococcaceae	Shin et al., 2015
Child day-care centers and outdoors (Edirne, Turkey)	Season, and RH and rain fall (negative correlation)	12 months	Season Temperature (°C) RH (%) Rainfall (mm) Wind speed (m/sn) Amount of sunlight (h/d) SO_2_ (μg/m^3^) PM (μg/m^3^) ^*^All centers were equipped with a central heating system, and ventilation was provided naturally through windows. ^*^People in the centers were not allowed to enter the rooms with their shoes on when going from outside to inside.	Air sampling Culture •Indoor (3-74 CFU/Petri plate^*^) •Outdoor (2-141 CFU/Petri plate^*^) ^*^ Collected using the Petri plate gravitational settling method (10 min for indoor air and 15 min for outdoor air)	Family *Indoor* Staphylococcaceae, Mycrococcaceae, Corynebacteriaceae, *Outdoor* Bacillaceae, Corynebacteriaceae, Staphylococcacea	Aydogdu et al., 2010
Hospital and outdoors (Internal wards, ICU, Outdoor)	Outdoor weather conditions (dust storms)	September–March	Building characteristics (size, number of beds) Number of patients and staff ^*^Windows and doors were closed during sampling.	Air sampling Culture •Internal wards (40–1,667) •ICU (27–605) •Outdoor (47–5,000)	Family *Indoor (internal wards and ICU)* Bacillaceae, Staphylococcaceae *Outdoor* Bacillaceae, Streptomycetaceae	Soleimani et al., 2016
Office^*^ and outdoor (^*^ Experiments were conducted in a controlled environmental chamber.)	Outdoors	December 9–20, (three times experiment/day, 2 h/experiment)	Cubature (m^3^) Area (m^2^) Ventilation system (HVAC) Temperature (°C) RH (%) Air exchange rate CO_2_ Number of persons Activities of persons	Air sampling Non-cultivation (MiSeq)	Family *Indoor* *Comamonadaceae, Flavobacteriaceae*, *Comamonadaceae* **Outdoor** *Comamonadaceae, Flavobacteriaceae, Corynebacteriaceae*	
Hospital and outdoor (patient rooms)	Outdoors: water and soil bacteria Indoors: human-associated bacteria (commensals or pathogens)	February 27–28	Temperature (°C) RH (%) Air flow rate ^*^Mechanically ventilated or naturally (window) ventilated room	Air sampling Non-cultivation (GS FLX Titanium)	*Phylum* *Indoor* Mechanically ventilated Betaproteobacteria, Alphaproteobacteria, Gammaproteobacteria Window-ventilated Betaproteobacteria, Alphaproteobacteria, Gammaproteobacteria, *Actinobacteria* *Outdoor* Betaproteobacteria, Alphaproteobacteria, Gammaproteobacteria, *Actinobacteria*	Kembel et al., 2012
**LAND USE**						
Urban and rural (urban: densely built-up environment and next to busy roads, rural: quiet, green and parklike residential area)	Land-use type	February 17	Traffic intensity NO PM_10_ PM_2.5_ Temperature (°C) RH (%)	Leaf sample (at least 200 cm^2^) Non-cultivation (MiSeq)	Family *Non-urban* Beijerinckiaceae, Methylocystaceae *Urban* Sphingomonadaceae, Flavobacteriaceae	Smets et al., 2016
Three land-use types (agricultural fields, suburban areas and forests) across northern Colorado, USA	Soils and leaf surfaces, fecal material, most likely dog faces	Two-week period during summer (sampled one site per day and rotated the order of collection between land-use types)	Land-use type Temperature (°C) RH (%) Dew point temperature Wind speed (km/h) Wind direction Barometric pressure (Mpa) Solar radiation (W/m^2^)	Air sampling Non-cultivation (454 GS FLX) Total bacterial concentrations^*^ 10^5^ −10^6^ cells/m^3^ ^*^Measured via flow cytometry	Phylum *Agricultural* Burkholderiales, *Actinobacteria*, *Firmicutes* *Suburban* *Actinobacteria*, Burkholderiales, *Firmicutes* *Forest* Burkholderiales, *FIrmicutes*, *Actinobacteria*, Rhizobiales	Bowers et al., 2011a

* Authors' comments or supplement information of each paper.

The relationship between indoor air quality and health has been researched since 1859, when open windows were found to be essential for maintaining healthy hospital rooms (Nightingale, 1999). Recently, NTM and *Legionella* infections have become problematic in developed countries. As NTM are ubiquitous in soil and water environments, whether infection can be attributed to the NTM discovered in a patient's home cannot be ascertained (Ichijo et al., 2014). Similarly, a study of *Legionella* infections in healthcare facilities in Italy identified the same *Legionella* serogroups in air and water samples, obscuring the true reservoir (Montagna et al., 2016). To clarify the sources of microorganisms causing infectious diseases, an integrated model is needed (see section Measured Factors at the Time of Airborne Bacterial Sampling in Built Environments).

Built environments are not closed systems, and allow the inflow and outflow of many materials. These spaces are also affected by humans and the outside air (Leung and Lee, 2016). Airborne transmission of microbes can follow different aerodynamic principles. Therefore, we must widen our focus and consider all parameters contributing to the maintenance of a well-balanced microbial community composition. Lymperopoulou et al., adopted the term meta-community to describe the microbial composition of the indoor air, which is influenced by microorganisms from environments surrounding the space in question (Lymperopoulou et al., 2016). Therefore, the living space, neighborhood, and whole city can be regarded as an ecosystem (a meta-community) (Figure 2).

## Measured factors at the time of airborne bacterial sampling in built environments

We propose measuring four factors (temperature, RH, air exchange rate, and occupant density) at the time of airborne bacterial sampling. However, this is controversial: some papers state that there is a correlation while others say there is none. In our review, we would like to show that these four factors are the current *minimum* requirement, since research on airborne bacteria in the built environment is ongoing. In studying a microbial community composition, researchers often measure various physicochemical values at the time of sampling (Tables 1, 2). The GSC provides a package for bacterial sequencing in a built environment (MIxS-BE: http://gensc.org/index.php?title=MIxS_extensions) which includes 26 metadata package terms (Glass et al., 2014; Ramos and Stephens, 2014). The influence of these physicochemical parameters on microbial communities has been investigated in soil and aquatic environments (Stres et al., 2008; Cole et al., 2013). It has also been studied in terms of fungal and bacterial growth in floor dust under elevated and continuous equilibrium relative humidity (ERH). This study indicated that the large increase in microbes at ERH levels >80% and the strong source terms of occupancy-driven resuspension may shape human exposure in buildings under continuous, elevated RH (Dannemiller et al., 2017). The correlation between RH and airborne bacteria in built environments has not been confirmed, although it presumed to influence bacterial growth (Chase et al., 2016). Lax et al. (2017) surveyed the bacterial diversity in a newly opened hospital and showed that the bacterial communities present on patients' skin strongly resembled those found in their room surfaces particularly on bedrails. They also analyzed the effect of environmental conditions on microbial transmission and found that microbial transmission was correlated with temperature, RH, and humidity ratio. It is not known whether transmission was caused by direct contact or via the air. A model that integrates the physicochemical factors related to transfer and migration, and the concentrations of bacteria in built environments could be useful in some situations (Zargar et al., 2016). In addition, devices that visualize airflow are now available, so the transfer of airborne microorganisms can be estimated by approximating the air exchange rate and volume of the inside and outside air.

In a hospital room maintained at constant temperature and RH (25°C and 55%, respectively), fungal and bacterial cells were identified from air samples for at least 3 days; however, by day 6, no fungal or bacterial colonies were obtained from the air samples (Moungthong et al., 2014). Although these studies cannot confirm whether a certain room temperature and RH suppress the growth of indoor bacteria, they do suggest that both factors control microbial communities. RH can affect the bacterial community, while its level of influence is different in indoor and outdoor spaces. Fierer et al. (2008), for example, found that indoor RH had no effect on the microbial community composition, but this composition was significantly correlated with humidity in outdoor air samples. Patient room airborne bacterial sampling conducted by Kembel et al. (2012) showed that building attributes, specifically the source of ventilation air, airflow rates, RH and temperature, were correlated with the diversity and composition of indoor bacterial communities. In the MTR samples, outdoor space and, the microbial diversity and/or abundance of certain genera were influenced by humidity, temperature, CO_2_ levels, and humans (Robertson et al., 2013; Leung et al., 2014; Afshinnekoo et al., 2015; Triado-Margarit et al., 2017). The influence of environmental factors (temperature, RH, air exchange rate, and occupant density) on bacterial abundance in the air has been reported by some studies, but a general relationship has not been confirmed. All of these observations support the proposal that microbiome data collection should be accompanied by the measurement of physicochemical factors, such as temperature, humidity, air exchange rate, and occupant density.

## Built environmental factors and airborne microbial legislation by country

Humans are constantly exposed to environmental microbes that can impact their microbiome. Here, we reviewed the sources of bacteria and the factors influencing the airborne bacterial communities and concentration in built environments. Bacteria in these spaces originate from different sources, and their communities are directly and indirectly affected by physical factors such as temperature and humidity. To clarify the factors influencing bacterial communities and concentration in human-occupied spaces, we must standardize the sampling and analysis protocols, as well as the physical parameters (temperature and humidity), as is already done by the Human Microbe Project (http://hmpdacc.org). It is now clear that fungal aerosols can cause human disease, and guidelines for fungi in indoor air have existed since 1979 (reviewed by Rao et al., 1996). Moreover, many countries, including the United States, Canada, and France, have established humidity standards for indoor environments because humidity significantly affects the growth of common fungi linked to allergies and breathing problems. China and South Korea have established air quality standards in buildings (Kim et al., 2003; Chan and Yao, 2008), and the American Industrial Hygiene Association has proposed guidelines outlining the safe maximum number of fungal spores in different indoor environments (http://www.wondermakers.com). Brazil, Hong Kong, and Singapore have already regulated the concentrations of airborne microorganisms in indoor environments. Given the health risks posed by airborne microorganisms, which are easily transmitted to different areas, it is important to note that the built environment equates to the sum total of all the assembled items that surround us, both natural and man-made. By understanding the effects of temperature, RH, air exchange rate, and occupant density on microbial communities in built-up areas, we can design healthier living spaces in future.

## Summary

Ramos and Stephens (2014), Glass et al. (2014), and other researchers in this field describe that there are major issues affecting the study of airborne bacteria in built environments, including difficulty in collecting data on the full set of the environmental parameters, and the absence of standardized protocols for data collection, analysis, and interpretation. To understand the links between airborne bacteria and various environmental parameters, as far as possible, the 26 parameters listed by MIxS-BE (http://gensc.org/index.php?title=MIxS_extensions) (see Figure 4) should be measured; however, the sampling situation, materials involved, and various other issues, mean that these measurements cannot always be taken. Therefore, in line with other researchers, we recommend the routine measurement of four environmental factors (temperature, RH, air exchange rate, and occupant density) to assess airborne bacteria in built environments, as a minimum requirement. By improving data collection, we can begin to understand the airborne bacteria environment of the built environment in more detail as a meta-community. This knowledge will provide insights into the relationship between humans and bacterial communities in this environment, and will help improve our (air) quality of life.

## Author contributions

SF drafted the manuscript, and DT and FM collected and reviewed the literature.

### Conflict of interest statement

The authors declare that the research was conducted in the absence of any commercial or financial relationships that could be construed as a potential conflict of interest.
